# Human Platelet Lysate Acts Synergistically With Laminin to Improve the Neurotrophic Effect of Human Adipose-Derived Stem Cells on Primary Neurons *in vitro*

**DOI:** 10.3389/fbioe.2021.658176

**Published:** 2021-03-19

**Authors:** Martino Guiotto, Wassim Raffoul, Andrew M. Hart, Mathis O. Riehle, Pietro G. di Summa

**Affiliations:** ^1^Department of Plastic, Reconstructive and Hand Surgery, Centre Hospitalier Universitaire Vaudois (CHUV), University of Lausanne (UNIL), Lausanne, Switzerland; ^2^Centre for the Cellular Microenvironment, University of Glasgow, Glasgow, United Kingdom; ^3^Canniesburn Plastic Surgery Unit, Glasgow Royal Infirmary, Glasgow, United Kingdom

**Keywords:** human platelet lysate, extracellular matrix, laminin, adipose-derived adult stem cells, serum substitute supplement, peripheral nerve repair, cell therapy

## Abstract

**Background:**

Despite the advancements in microsurgical techniques and noteworthy research in the last decade, peripheral nerve lesions have still weak functional outcomes in current clinical practice. However, cell transplantation of human adipose-derived stem cells (hADSC) in a bioengineered conduit has shown promising results in animal studies. Human platelet lysate (hPL) has been adopted to avoid fetal bovine serum (FBS) in consideration of the biosafety concerns inherent with the use of animal-derived products in tissue processing and cell culture steps for translational purposes. In this work, we investigate how the interplay between hPL-expanded hADSC (hADSC^hPL^) and extracellular matrix (ECM) proteins influences key elements of nerve regeneration.

**Methods:**

hADSC were seeded on different ECM coatings (laminin, LN; fibronectin, FN) in hPL (or FBS)-supplemented medium and co-cultured with primary dorsal root ganglion (DRG) to establish the intrinsic effects of cell–ECM contact on neural outgrowth. Co-cultures were performed “direct,” where neural cells were seeded in contact with hADSC expanded on ECM-coated substrates (*contact effect*), or “indirect,” where DRG was treated with their conditioned medium (*secretome effect*). Brain-derived nerve factor (BDNF) levels were quantified. Tissue culture plastic (TCPS) was used as the control substrate in all the experiments.

**Results:**

hPL as supplement alone did not promote higher neurite elongation than FBS when combined with DRG on ECM substrates. However, in the presence of hADSC, hPL could dramatically enhance the stem cell effect with increased DRG neurite outgrowth when compared with FBS conditions, regardless of the ECM coating (in both indirect and direct co-cultures). The role of ECM substrates in influencing neurite outgrowth was less evident in the FBS conditions, while it was significantly amplified in the presence of hPL, showing better neural elongation in LN conditions when compared with FN and TCPS. Concerning hADSC growth factor secretion, ELISA showed significantly higher concentrations of BDNF when cells were expanded in hPL compared with FBS-added medium, without significant differences between cells cultured on the different ECM substrates.

**Conclusion:**

The data suggest how hADSC grown on LN and supplemented with hPL could be active and prone to support neuron–matrix interactions. hPL enhanced hADSC effects by increasing both proliferation and neurotrophic properties, including BDNF release.

## Introduction

Peripheral nerve injuries (PNI), despite advancements of microsurgical techniques, lead to a profound reduction in patients’ quality of life and pose a socioeconomic burden. Commonly, PNI result from trauma, tumor extirpation, or iatrogenic injuries to the upper and lower limb, mainly affecting young and adult working age classes.

Unlike the central nervous system, the peripheral nervous system (PNS) retains a spontaneous capacity to regenerate over short gaps, due to the plasticity of Schwann cells (SCs).

Currently, the gold-standard treatment is an end-to-end anastomosis in the case of short gap, while nerve grafts are generally chosen for long segment nerve defects (>2–3 cm) because of the otherwise excessive tension between the two stumps. These procedures lead to unsatisfactory outcomes and suboptimal functional recovery, followed by consequent donor site morbidity in the latter surgical option. Poor outcomes can be explained by the chronic denervation and fibrosis of the distal stump which rapidly affects the regenerative capability of the PNS (Jessen and Mirsky, 2016).

Tissue engineering pursues to find the ideal biodegradable conduit, in order to avoid the common nerve graft complications, preserving or even improving the functional outcomes in clinical care. In the last decade, the concept of an empty conduit was overcome by the application of intraluminal coatings or fillers containing extracellular matrix (ECM) molecules [laminin (LN), fibronectin (FN), or collagen], transplanted cells [SCs, SC-like cells, adipose-derived stem cell (ADSC)], and neurotrophic factors [mainly brain-derived nerve factor (BDNF), glial-derived nerve factor (GDNF), nerve growth factor (NGF)], which improve the recovery in the PNS (Resch et al., 2018).

The ideal cell type should be easily transplantable with no associated functional donor loss, proliferate rapidly in *in vitro* expansion, and successfully integrate into host tissue with minimal immunological reaction. Previous experimental studies in rodent have shown that ADSC transplantation represents effectively an alternative strategy to create a favorable environment for nerve regeneration without the drawbacks of SCs (need for nerve biopsy and sacrifice of a functional nerve, constant need for GF adjunction and more complex *in vitro* expansion) (Kingham et al., 2014; Mathot et al., 2019).

The growing interest in stem cell therapy in a wide range of medical fields opened new lines of investigation for nerve repair, with a particular focus on human ADSC (Lensch et al., 2018).

To achieve safe and reliable clinical translation, stem cell therapy has to be subjected to biosafety concerns (Paula et al., 2015; Condé-Green et al., 2016). The manipulation with xenogeneic components, including enzymatic adipose tissue dissociation and the use of fetal bovine serum (FBS) as culture medium supplement, showed the potential of immune reactions and exposure to viral, bacterial, or prion infection (Lalu et al., 2012; Sherman et al., 2019). Moreover, FBS is subject to batch-to-batch variability, which impacts on reproducibility. According to the good manufacturing practice (GMP) guidelines, the essential steps to ensure translationability for stem cell therapy are the avoidance of chemical- and/or animal-derived protein in tissue dissociation and culture supplementation (Kyllönen et al., 2013; Condé-Green et al., 2016).

According to these considerations, human platelet lysate (hPL) was suggested as a substitute to FBS for human cell expansion assuring the biosafety and consistent reliability in clinical translation, since it can be easily obtained, as pooled blood from apheresis products and buffy coats (Schallmoser et al., 2020).

Therefore, human adipose-derived stem cells (hADSC) expanded in hPL-supplemented medium (hADSC^hPL^) can be seen as a viable cell population for therapy in nerve defects, with translational applicability and neurotrophic potential (Palombella et al., 2020).

Regarding a further potential actor involved in PNS regeneration, the ECM, an acellular component composed of proteoglycans and fibrous proteins such as collagen, elastin, fibronectin, and laminin, provides a well-defined environment for cell survival, differentiation, tissue morphogenesis, and homeostasis (Frantz et al., 2010; de Luca et al., 2014). LN is a main component of the ECM both in the central and the peripheral nervous systems, supporting a variety of functions including SC migration, axonal outgrowth, and axon myelination (Barros et al., 2011; di Summa et al., 2013). FN is secreted by glial cells promoting cell growth, survival, and motility. Both support the recovery after nerve injuries: the former (LN) stimulating axonal elongation and activating SCs in myelin production, the latter (FN) increasing neural cell adhesion and SC proliferation (Gao et al., 2013).

After a nerve lesion in PNS, SCs dedifferentiate into a “repair” SC phenotype, migrate, proliferate along the endothelial bridge across the nerve gap, secrete growth factors, support axonal elongation, and myelinate axons. The ECM provides the binding sites mediating interactions between cells, growth cone, and the surrounding microenvironment through specific integrin receptors, which regulate cell adhesion, proliferation, migration, and differentiation (Plow et al., 2000; Schmidt and Leach, 2003; Scheib and Höke, 2013). While being a critical component for cell-to-cell interactions, ECM also acts as a reservoir for soluble factors bound to its components through specific receptors (Harris et al., 2017).

By providing the cells with specific ECM cues, it is possible to influence and improve the regenerative process and gain further insights into its therapeutic potential.

Building on our previous works, and in order to define the most suitable and encouraging coating for a future bioconduit, we investigated the impact of hADSC^hPL^ in combination with different ECM-molecule-coated surfaces, and we studied their individual and combined potentials to improve neurite outgrowth in a dorsal root ganglia (DRG) explant model.

## Materials and Methods

### hADSC Extraction and Culture

hADSC were isolated from abdominal adipose tissue of three healthy women (age 49 ± 2) who underwent breast reconstruction using abdominal autologous flaps (deep inferior epigastric perforator flaps, DIEP) at Canniesburn Plastic Surgery Unit, Glasgow Royal Infirmary, Glasgow, Scotland. The discarded part of the flap and the adipose tissue were obtained after a signed informed consent. All protocols were reviewed and approved by the hospital ethics committees (local Biobank number 314 GGC) in accordance with the Declaration of Helsinki.

The adipose tissue was mechanically minced, microdissected, and mechanically dispersed in a 10-cm Petri dish, until the hADSC were selected according to their plastic adherence. They were resuspended in 7 ml of complete medium (see below for composition), plated in T75 flask, and cultured at 37°C and 5% CO_2_. The medium was changed after 24 h to remove erythrocytes and, afterward, every 3–4 days until cell confluence.

Cells isolated were cultured in parallel in complete medium: alpha-MEM (Gibco, Paisley, United Kingdom) added with 5% hPL (Antibodies, Aachen, Germany) or 10% FBS (Sigma-Aldrich, United Kingdom) and 1% penicillin–streptomycin (GE Healthcare).

### Extracellular Matrix Surface Preparation and Cell Seeding

Tissue culture plastic (TCPS) 24-well plates were use direct or coated according to the specific condition with either LN (LN-1, Engelbreth-Holm-Swarm murine sarcoma basement membrane, Sigma-Aldrich) for 2 h at 37°C at 10 μg/ml phosphate buffered saline (PBS) or FN (bovine plasma, Sigma-Aldrich) at 10 μg/ml PBS for 1 h at room temperature. For the control, TCPS well surface was used without any prior coating. Every coating step was followed by a double washing with PBS 1 × solution (Sigma-Aldrich). The concentration of the ECM proteins and the timing for coating procedures were based on our previous experience (di Summa et al., 2013; de Luca et al., 2018).

hADSC were trypsinized and homogeneously seeded at a density of 15,000 cells/cm^2^ in 24-well plates, incubated for 48 h at 37°C, 5% CO_2_, and then seeded with freshly dissected DRG.

### DRG Organotypic Explants

All work was carried out in accordance with the Home Office Animals (Scientific Procedures) Act 1986. Neonatal Sprague–Dawley rats (1–3 days old) were euthanized by Euthatal^®^ injection (Merial, 200 mg/ml, 500 mg/kg), carried out by an animal house technician in accordance with Home Office regulations, and DRG carefully dissected using the microsurgical technique under binocular magnification. Upon extraction, DRG were transferred to an ice-cooled Petri dish with Leibovitz’s L-15 Medium (Thermo Fisher Scientific, Loughborough, United Kingdom) and processed to remove excess roots. DRG organotypic explants were, after delicate microsurgical root cleaning, immediately cultured (one single DRG/well) under the three conditions detailed below. After 48 h of co-culture, DRG were fixed and stained following the protocol below.

### hADSC–DRG Co-Cultures

With the co-cultures, we wanted to reproduce *in vitro* the supposed *in vivo* effects of hADSC when used in a nerve conduit for peripheral nerve repair. We adapted the co-culture model to investigate the interactions between ECM proteins (condition 1), hADSC (condition 2), and hADSC secretome only (condition 3) with primary neurons. Considering the *in vivo* timescale of nerve repair, these three conditions could mimic the spatial–temporal contacts of the growth cone after the implantation of a cell-filled nerve conduit at the injury site (Table 1).

**TABLE 1:** DRG explant co-culture *in vitro* model to mimic the hPL–hADSC therapy on ECM-coated scaffold *in vivo*.

	**Cells in culture**	**Culture medium**	**Substrate**	***In vivo* interaction simulation**
Condition 1—DRG alone	Primary DRG explant	50% DRG medium, 50% hADSC medium	24-well plate coated with LN, FN, or TCPS	Growth cone and ECM
Condition 2—direct co-culture	Primary DRG explant and hADSC	50% DRG medium, 50% hADSC medium Serum-free condition: 50% DRG medium, 50% hADSC medium (without 5% hPL or 10% FBS)	24-well plate coated with LN, FN, or TCPS	Growth cone, ECM, and hADSC (cells + secretome)
Condition 3—indirect co-culture	Primary DRG explant	50% DRG medium, 50% hADSC secretome	24-well plate coated with LN, FN, or TCPS	Growth cone, ECM, and hADSC (secretome)

*hPL, human platelet lysate; hADSC, human adipose-derived stem cells; ECM, extracellular matrix; DRG, dorsal root ganglion; LN, laminin; FN, fibronectin; TCPS, tissue culture plastic.*

#### Condition 1 (DRG Alone)

Single DRG was grown on a 24-well plate coated with LN, FN, or TCPS only for 48 h at 37°C, 5% CO_2_ in a mix of 100 μl of L-15 media (Thermo Fisher), 50 μg/ml *N*-acetylcysteine (NAC, Sigma-Aldrich) (West et al., 2007), 1% penicillin–streptomycin (Pen-Strep, GE Healthcare), 10 ng/ml NGF 2.5S (Invitrogen) (*DRG medium*), and 100 μl of Alpha-MEM (Gibco) with 5% hPL (Antibodies) or 10% FBS (Sigma-Aldrich) (*hADSC medium*). To make this condition comparable with the following ones, the co-culture medium was prepared by mixing in equal parts the DRG medium with complete hADSC medium.

#### Condition 2 (Direct Co-Cultures)

A single DRG was cultured on top of hADSC (15,000/cm^2^ in a 24-well plate pre-expanded for 48 h on LN, FN-coated surface, or TCPS at 37°C, 5% CO_2_) in a mixture of 100 μl of DRG medium and 100 μl of hADSC medium for 48 h at 37°C, 5% CO_2_. The same direct co-cultures were repeated in parallel without medium supplementation with FBS or hPL (*serum-free*) but keeping these additives only during hADSC expansion steps (to closely mimic the *in vivo* condition, Table 1).

#### Condition 3 (Indirect Co-Cultures)

**FIGURE 1:**
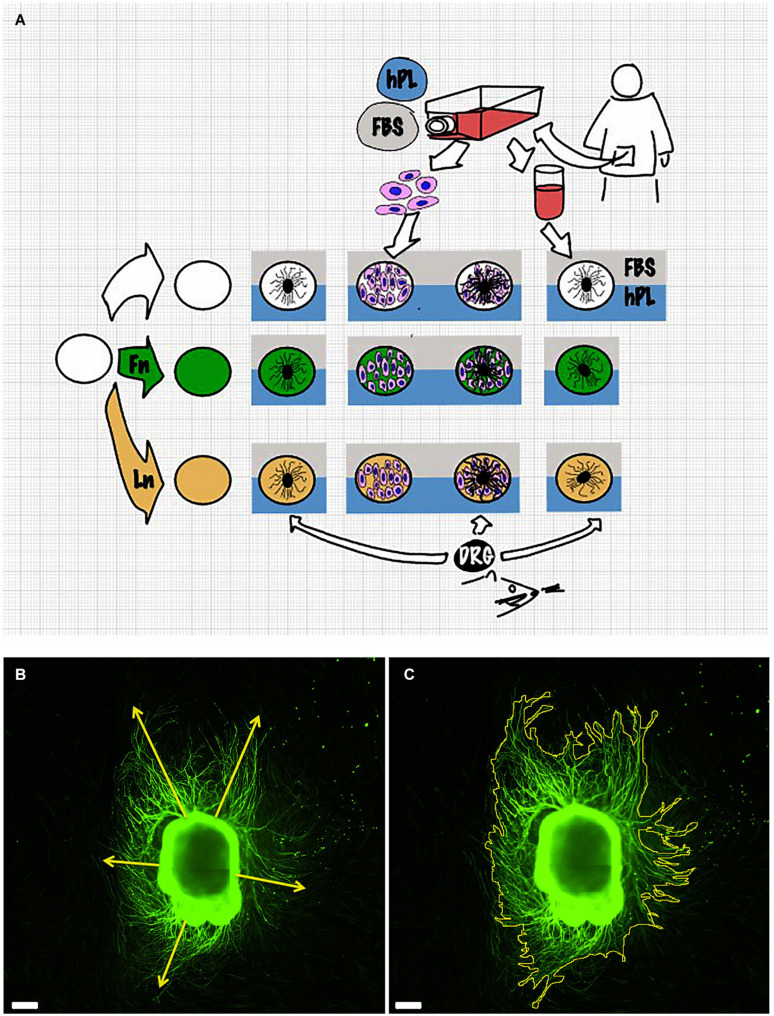
Scheme of the experimental setup **(A)**. Details of the measurement method: dorsal root ganglion (DRG) longest neurite **(B)** and area **(C)**. Scale bar: 500 μm.

A single DRG explant was cultured on a 24-well plate coated with either LN, FN, or TCPS for 48 h at 37°C, 5% CO_2_ in a mixture of 100 μl of DRG medium and 100 μl of conditioned medium of the hADSC (*secretome*) previously grown (initial same cell density, 15,000/cm^2^) on a substrate coated with either LN, FN, or TCPS for 48 h in the presence of hADSC complete medium (Figure 1A).

### Immunofluorescence

After 48 h, DRG were fixed and stained with mouse anti-β3-tubulin (Sigma-Aldrich Tub III, 1:100) and FITC-coupled phalloidin (Thermo Fisher; 1:200) followed by Texas Red-labeled antimouse (1:100, Vector Laboratories, United States) and DAPI (VECTASHIELD, Vector Laboratories, Peterborough, United Kingdom).

### Image Analysis

Samples were viewed on an Olympus BX51 fluorescent microscope using 10 × (Olympus, NA 0.3). Images were acquired using a high-resolution camera (QImaging, Retiga 2000i), an automated stage movement (Prior Scientific, United Kingdom) combined with the auto-stitching of mosaic images with Surveyor Viewer software (Objective Imaging, United States).

DRG scanning images (anti-β3-tubulin channel) were analyzed in Fiji ImageJ software (Schindelin et al., 2012).

Both image capturing and neurite outgrowth analysis were performed by the same examiner. Results were expressed as the longest neurite and “axonal area,” which was defined as the area that was covered by β3-tubulin-positive neurites. This was used as the whole extension of the DRG and its outgrowth was imaged for each condition. The neurite length was manually evaluated using the Freehand line tool after setting the appropriate scale for calibration. For every picture, at least five different attempts were performed to identify the longest neurite. The area was identified with the Freehand area tool following the perimeter of each neurites. Experiments were repeated for three biological repeats and at least three technical repeats for each condition (Figures 1B,C).

### Evaluation of BDNF-Secreted Factor

The conditioned medium was collected after expansion of hADSC for 48 h on coated (LN or FN) surfaces or on uncoated TCPS in the presence of either of the two supplements (FBS, hPL). After centrifugation step at 4,300 relative centrifugal force for 10 min to remove cellular debris, supernatants were transferred to a new tube and stored at −20°C until processing. Fresh non-conditioned medium was used as negative control. Subsequently, samples were analyzed with ELISA kits for BDNF (R&D Systems, Bio-Techne DuoSet ELISA Human BDNF, Abingdon, United Kingdom), according to the manufacturer’s instruction. Each sample was assayed in duplicate and the absorbance determined at 450 and 570 nm with an infinite F50 spectrophotometer (Tecan Group, Männedorf, Switzerland). The quantity of the secreted factor, calculated from a standard curve produced using recombinant protein, was multiplied with the dilution factor. Experiments were conducted in technical and biological triplicates.

### Statistical Analysis

All data were expressed as average ± standard error of the mean (SEM). To verify the data spread, D’Agostino and Pearson omnibus normality test was applied (D’Agostino, 1971).

One-way analysis of variance (ANOVA) with Tukey’s multiple comparison test was used to assess statistical significance among groups.

Significance was expressed as ^∗^*p* < 0.05, ^∗∗^*p* < 0.01, ^∗∗∗^*p* < 0.001, and ^****^p < 0.0001. All analysis was performed using GraphPad Prism 6 for Mac (GraphPad Software, La Jolla, CA, United States).

## Results

### Serum Additives Significantly Influence DRG Neurite Outgrowth but Only in Combination With Laminin

To understand the impact of hPL on primary neurons, DRG explants were cultured on LN or FN-coated TCPS (with uncoated TCPS used as a negative control) for a time period of 48 h. The hypothesis that a higher concentration of trophic molecules present in hPL may enhance nerve regeneration on their own was not confirmed: DRG outgrowth, both in terms of the longest neurite as well as axonal area, was comparable regardless of the serum supplement (FBS or hPL) adopted for the medium.

**FIGURE 2:**
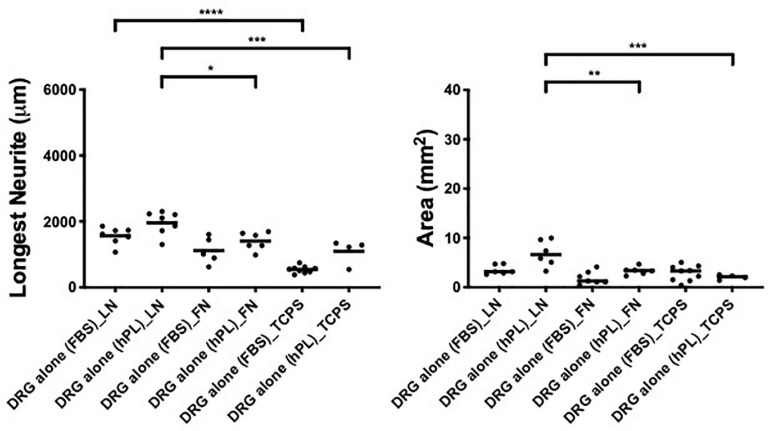
Serum additives significantly influence DRG neurite outgrowth, but only in combination with laminin (condition 1—DRG alone). Longest neurite and axonal area of DRG alone on the extracellular matrix (ECM). Three biological repeats and at least three technical replicates (DRG) were assessed. One-way ANOVA with Tukey’s multiple comparison tests was used for assessing statistical significance among the examined groups (*p < 0.05, **p < 0.01, ***p < 0.001, ****p < 0.0001). The number of DRG tested corresponds to the number of dots for each condition in the graph.

However, when considering the ECM impact on DRG outgrowth, our results showed meaningful variation when DRG were cultured on LN-coated surfaces. This effect was visible in both DRG in media supplemented with either hPL or FBS in terms of neurite extension [LN vs TCPS (FBS), ^****^p < 0.0001; LN vs TCPS (hPL), ^∗∗∗^*p* < 0.001; Figure 2], but only in hPL conditions when considering axonal area [LN vs TCPS (hPL), ^∗∗∗^*p* < 0.001]. Similarly, LN enhanced neurite elongation significantly more so than FN, but this effect was statistically significant only for hPL (LN vs FN: longest neurite, ^∗^*p* < 0.05; axonal area, ^∗∗^*p* < 0.01; Figure 2).

### ECM, hADSC, and hPL Synergistically Enhance DRG Outgrowth

To assess the effect of hADSC on neural cells, comparing FBS to when hPL is applied as the medium additive in the pre-expansion steps (hADSC^FBS^, hADSC^hPL^), we performed a direct co-culture of hADSC^FBS^ or hADSC^hPL^ with single DRG on LN, FN-coated surface, or TCPS.

hPL and FBS differ considerably in their content (growth factors, adhesion factors, cytokines, and bulk protein), which may directly influence DRG explant outgrowth; therefore, the hADSC were only precultured with either media, then continued in serum-free conditions once the DRG were added. These experiments were performed in parallel to the same direct co-cultures (with serum additives) between hADSC and primary neurons.

The DRG explant outgrowth was the same, and no significant difference was found between serum-free and serum-added groups, independently of the ECM (Appendix 1), confirming that the medium additives themselves did not influence DRG outgrowth directly. Applying hPL only during the expansion steps of hADSC was sufficient to obtain a significant neurotrophic effect on neurons, which was consistent with our previous results (Palombella et al., 2020).

**FIGURE 3:**
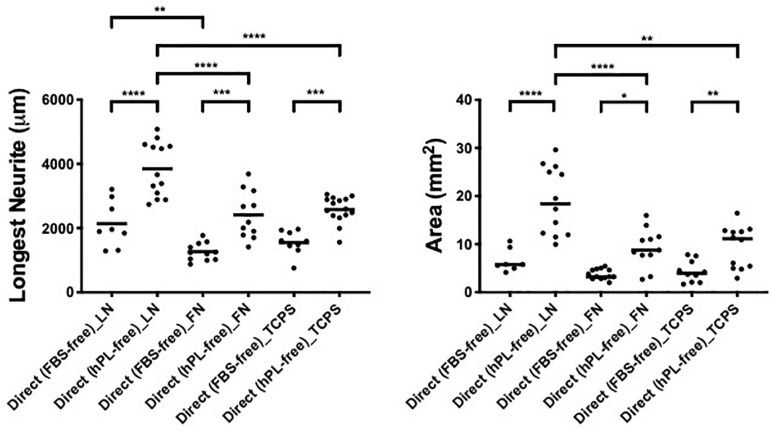
ECM, human adipose-derived stem cells (hADSC), and human platelet lysate (hPL) synergistically interact enhancing DRG outgrowth (condition 2—direct co-culture). Longest neurite and axonal area of direct co-culture on ECM. Three biological repeats and at least three technical replicates (DRG) were assessed. One-way ANOVA with Tukey’s multiple comparison tests was used for assessing statistical significance among the examined groups (*p < 0.05, **p < 0.01, ***p < 0.001, ****p < 0.0001). The number of DRG tested corresponds to the number of dots for each condition in the graph.

The DRG longest neurite was strongly increased by hADSC^hPL^ in all conditions, compared with the same co-culture system with hADSC^FBS^ (^****^p < 0.0001 on LN, ^∗∗∗^*p* < 0.001 on FN, ^∗∗∗^*p* < 0.001 on TCPS, Figure 3). Similarly, the axonal area was higher in hADSC^hPL^ conditions on LN (^****^p < 0.0001), FN (^∗^*p* < 0.05), and TCPS (^∗∗^*p* < 0.001) when compared with the ADSC^FBS^ (Figure 3).

Importantly, the hADSC^hPL^ cultured on LN lead to a statistically stronger DRG outgrowth (both the longest neurite and axonal area) compared with any other condition, including versus hADSC^hPL^ on FN (longest neurite and area: ^****^p < 0.0001) or TCPS (longest neurite ^****^p < 0.0001; area ^∗∗^*p* < 0.01). The differences in DRG explant outgrowth between the different ECM coatings were less pronounced in any of the FBS conditions. Although DRG explants on ADSC^FBS^ cultured on LN showed a significantly increased neurite outgrowth when compared with FN, this was not the case when compared with the outgrowth seen on ADSC^FBS^ on TCPS (Figure 3).

### hADSC^hPL^ Secretome Supports DRG Outgrowth With High Affinity for ECM Molecules

To analyze the impact of the hADSC secretome alone on DRG outgrowth, without the effect of cell-to-cell interactions, we realized an indirect co-culture model with single DRG grown on LN, FN-coated surface, or TCPS and treated with hADSC-conditioned medium that was derived from hADSC cultures supplemented either with FBS or with hPL, cultured on TCPS alone, or TCPS coated with either LN or FN.

**FIGURE 4:**
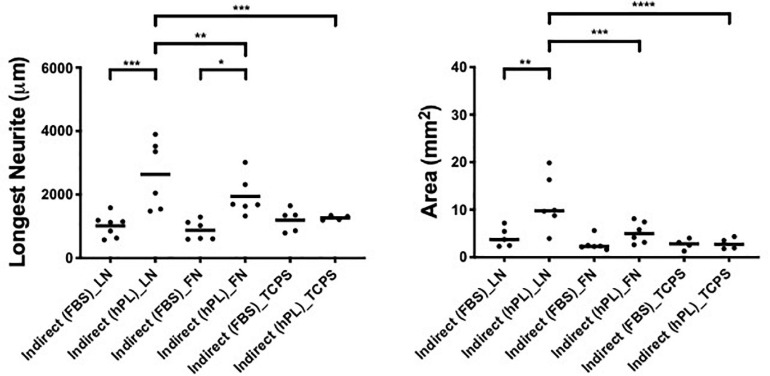
hPL–hADSC secretome supports DRG outgrowth with high affinity for ECM molecules (condition 3—indirect co-cultures). Longest neurite and axonal area of indirect co-culture on ECM. Three biological repeats and at least three technical replicates (DRG) were assessed. One-way ANOVA with Tukey’s multiple comparison tests was used for assessing statistical significance among the examined groups (*p < 0.05, **p < 0.01, ***p < 0.001, ****p < 0.0001). The number of DRG tested corresponds to the number of dots for each condition in the graph.

The DRG outgrowth (both the longest neurite and axonal area) was significantly increased by the hADSC^hPL^ secretome (vs ADSC^FBS^ secretome) on either of the two ECM substrates (longest neurite: LN ^∗∗∗^*p* < 0.001, FN ^∗^*p* < 0.05; axonal area: LN ^∗∗^*p* < 0.01) but not on TCPS (Figure 4).

The strongest neurotrophic effect was seen on DRG explants placed on LN and stimulated by hADSC^hPL^ secretome. The hADSC^hPL^ secretome evoked the longest neurite to grow of DRG explants and lead to axonal areas that were significantly higher than on either FN or TCPS (longest neurite: vs FN ^∗∗^*p* < 0.01, vs TCPS ^∗∗∗^*p* < 0.001; area: vs FN ^∗∗∗^*p* < 0.001, vs TCPS ^****^p < 0.0001).

The ADSC^FBS^ secretome showed no synergistic effects in combination with the different substrate types on axonal outgrowth of DRG (Figure 4).

### Influence of ECM Molecules on DRG Outgrowth

**FIGURE 5:**
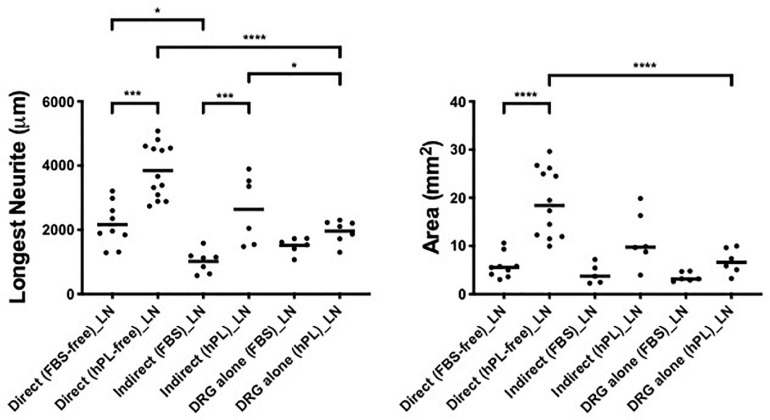
Influence of laminin on DRG outgrowth. Longest neurite and axonal area of direct co-culture, indirect co-culture, and DRG alone on laminin (LN). Three biological repeats and at least three technical replicates (DRG) were assessed. One-way ANOVA with Tukey’s multiple comparison tests was used for assessing statistical significance among the examined groups (*p < 0.05, **p < 0.01, ***p < 0.001, ****p < 0.0001). The number of DRG tested corresponds to the number of dots for each condition in the graph.

The combination of a direct co-culture of the DRG explants on hADSC grown with hPL and on LN showed the most effective approach *in vitro* for nerve regeneration; this combination showed the longest DRG neurite outgrowth (almost 4,000 μm) and the largest axonal area (almost 20 mm^2^). For the indirect co-culture, the secretome of hADSC^hPL^ on LN resulted in a significantly higher axonal elongation (^∗^*p* < 0.05) when compared with the DRG explant on its own on LN (Figure 5).

**FIGURE 6:**
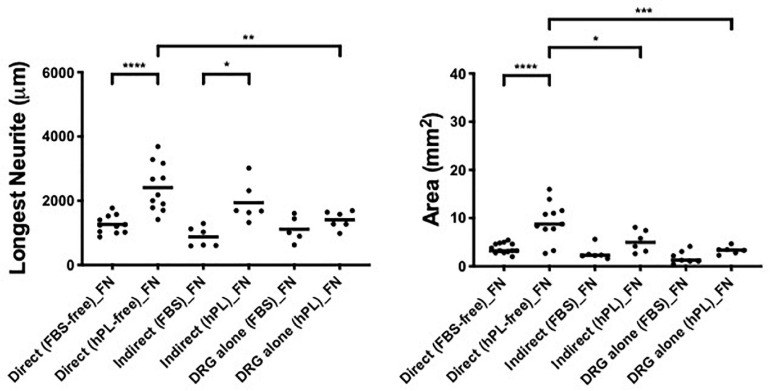
Influence of fibronectin on DRG outgrowth. Longest neurite and axonal area of direct co-culture, indirect co-culture, and DRG alone on fibronectin (FN). Three biological repeats and at least three technical replicates (DRG) were assessed. One-way ANOVA with Tukey’s multiple comparison tests was used for assessing statistical significance among the examined groups (*p < 0.05, **p < 0.01, ***p < 0.001, ****p < 0.0001). The number of DRG tested corresponds to the number of dots for each condition in the graph.

hADSC^hPL^ grown on FN-coated surfaces showed, once in contact with DRG explants, a beneficial effect on neurite outgrowth, when compared with the DRG alone on FN, even if with significantly lower values for the longest neurite or axonal area when compared with laminin-coated substrates (direct vs DRG alone, longest neurite: ^∗∗^*p* < 0.01, axonal area: ^∗∗∗^*p* < 0.001; direct vs indirect, area ^∗^*p* < 0.05, Figure 6).

Interestingly, the positive effect of hADSC grown on FN was only visible in the direct co-culture system and only in the presence of hPL. When the secretome of hADSC grown on FN or TCPS was used as a culture medium (condition 2), DRG explant outgrowth was not significantly impacted when compared with the DRG explant grown directly on the respective ECM surface.

**FIGURE 7:**
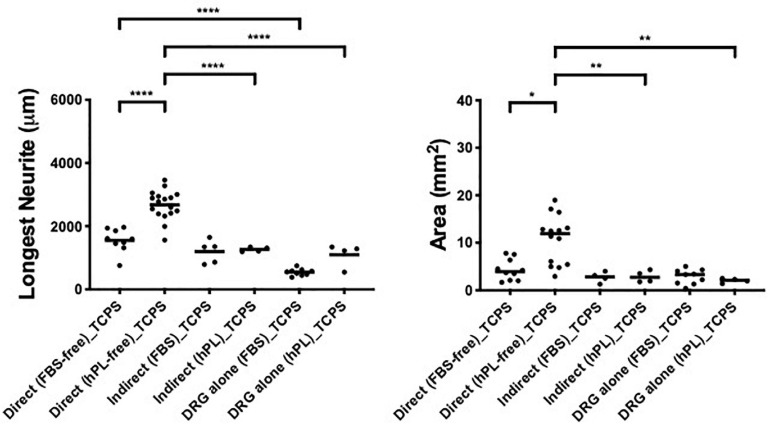
Influence of tissue culture plastic (TCPS) on DRG outgrowth. Longest neurite and axonal area of direct co-culture, indirect co-culture, and DRG alone on TCPS. Three biological repeats and at least three technical replicates (DRG) were assessed. One-way ANOVA with Tukey’s multiple comparison tests was used for assessing statistical significance among the examined groups (*p < 0.05, **p < 0.01, ***p < 0.001, ****p < 0.0001). The number of DRG tested corresponds to the number of dots for each condition in the graph.

hADSC^hPL^ grown on TCPS evidenced a neurotrophic effect only when the primary DRG explants were in direct contact with the hADSC (longest neurite: vs indirect ^****^ < 0.0001, vs DRG explant alone ^****^ < 0.0001; area: vs indirect ^∗∗^ < 0.01, vs DRG explant alone ^∗∗^ < 0.01, Figure 7).

Here, the direct co-culture appeared significantly better than DRG explant alone even when FBS was used as additive medium (longest neurite: ^****^ < 0.0001, Figure 7).

### hADSC^hPL^ Show Enhanced BDNF Secretion

We evaluated the secretion of BDNF in hADSC-conditioned medium collected after 48 h of cell expansion on LN, FN, or TCPS with medium supplemented either with 5% hPL or 10% FBS.

**FIGURE 8:**
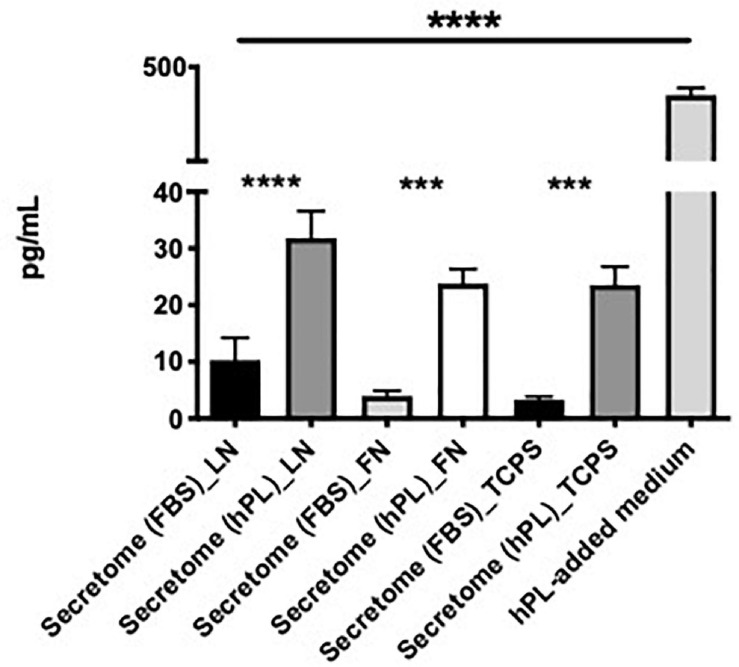
hPL–hADSC show enhanced brain-derived nerve factor (BDNF) secretion. Evaluation of BDNF concentration (pg/ml) in hADSC-conditioned medium (secretome) on LN, FN, or TCPS. Both supplement media (FBS and hPL) were tested and compared according to the ECM-coating condition. BDNF concentration was evaluated in hPL-added medium alone (without cells), too. Three biological and technical (each one in duplicate) repeats were assessed. One-way ANOVA with Tukey’s multiple comparison tests was used for assessing statistical significance among the examined groups (*p < 0.05, **p < 0.01, ***p < 0.001, ****p < 0.0001).

The hPL alone had a significantly higher concentration of BDNF when compared with the hADSC-conditioned medium, regardless of the ECM-coating options. In all three ECM-coating conditions, the levels of BDNF detected in the 5% hPL-added medium were significantly increased compared with the media supplemented with 10% FBS (^****^p < 0.0001, Figure 8).

The hADSC cultured under serum-free conditions did not show a significantly different change in BDNF release independently of the ECM substrate that they grew on. When focusing on a potential ECM effect con the GF release, hADSC^hPL^ on LN did not seem to result in any significant changes to the free BDNF levels in the supernatant (Figure 8).

## Discussion

PNI can cause motor, sensory, and autonomic disabilities. Despite the spontaneous regeneration of the peripheral nervous system and microsurgical advancements in nerve repair, the outcomes remain far from optimal, with unsatisfactory functional results and high morbidity (Xu et al., 2019). The necessity of ameliorating the restorative potential has guided research interest toward cellular therapies, which aim to use autologous cells to support the host regeneration process (Guo et al., 2017).

hADSC have attracted interest for their cell-based therapy potential because of their abundance, ease of isolation, high proliferation rate, greater differentiation potential compared with other mesenchymal stem cells (MSCs), and their immunomodulatory and angiogenic properties (Kim et al., 2013; Palombella et al., 2017; Cowper et al., 2019). The regenerative capability is the synergy between the cell-to-cell interactions and what cells release in the surrounded milieu (Teixeira et al., 2015). Thus, recent cell therapy research has been focused on the potential applications of the MSC secretome due to its GF-enriched composition and extracellular vesicles. Nevertheless, the proteomic studies are still insufficient to explain the functional effects of the MSC secretome, and to specify the diverse protein spectra according to the type of MSCs, some studies showed an increased neuronal and glial survival rate or a more pronounced neural/glial differentiation, when neural precursor cells were cultured with human umbilical cord perivascular cell (HUCPVC) secretome, possibly explained by the release of NGF, fibroblast growth factor-2 (FGF-2), or intracellular protein such as 14-3-3, Hsp70, and UCHL1 (Ribeiro et al., 2012; Teixeira et al., 2015).

Recently, we developed a completely animal protein-free method to extract, culture, and maintain *in vitro* hADSC, guaranteeing patient biosafety and potential for clinical translation (Palombella et al., 2020).

As a medium additive, hPL provides a safe, human-derived product, which has been shown to increase the proliferation of hADSC, preserve their stemness properties and differentiation potential, and even enhance their neural commitment (Guiotto et al., 2020; Lischer et al., 2020; Palombella et al., 2020). For all these reasons, hADSC pre-expanded in hPL (hADSC^hPL^) are the ideal candidate for cell-based therapies and can play a role in nerve tissue engineering encouraging regeneration.

Functionalization of biomaterial surfaces to influence cellular behavior can be a fruitful approach to deliver and maintain transplanted cells for medical application. Such cells may be induced toward target cells by reproducing the original regeneration environment through physical and biochemical components (Zhang et al., 2019).

In this sense, when considering the interplay between the ECM and neural cells, the design of a hADSC^hPL^ cell delivery scaffold needs to simulate the physical and haptotactic cues of the ECM, guiding the axonal elongation across the nerve stumps, through both soluble signaling and contact integrin receptor pathways (Chen et al., 2000; Armstrong et al., 2007; Dodla and Bellamkonda, 2008; Guo et al., 2015, 2017; Poitelon et al., 2016).

According to the literature, ECM components (FN, LN, and collagen) have been successfully applied as inner coating of neural conduits in rodent *in vivo* models, successfully increasing SC and MSC proliferation, cell viability, and functional outcomes (Armstrong et al., 2007; Gámez Sazo et al., 2012; de Luca et al., 2013; Gonzalez-Perez et al., 2018). LN has been reported to be an important actor when considering ECM proteins that have been shown to promote nerve outgrowth, being essential for SC proliferation, morphological/phenotypical modifications, and myelination (Milner et al., 1997; Chernousov et al., 2008; Yu et al., 2009; Koh et al., 2010; di Summa et al., 2013; Hosseinkhani et al., 2013; de Luca et al., 2014).

Interestingly, LN impacts consistently not only on neural cell growth but also on ADSC attachment, survival, and SC-like differentiation. Our group previously showed that LN better supported rat ADSC differentiated toward a glia-like phenotype (dADSC) adhesion and survival and in co-culture with neurons supporting an increased neurite length. Consistently, we detected a boost to growth factor secretion (NGF and BDNF) when dADSC were grown on ECM (LN or FN) with DRG (di Summa et al., 2013).

Close to our outcomes, Zarinfard et al. (2016) found that human adipose cells transdifferentiated into SC-like, grown on LN surface exhibited higher cell viability and stronger gene expression of GFAP, S100-Beta, myelin basic protein (MBP), and even BDNF and GDNF.

Gonzalez-Perez et al. showed better *in vitro* proliferation and cell survival of SCs when cultured in LN than in FN. However, in their *in vivo* study, with a critical rat sciatic nerve gap, SCs seeded in FN-aligned matrices showed superior functional results than SCs loaded in LN-aligned matrices. Regarding the application of MSCs (derived from bone marrow) in the same aligned constructs, better outcomes were reported both *in vitro* and *in vivo*, when surfaces were coated with FN (Gonzalez-Perez et al., 2018).

With translation to the clinic in mind, and to avoid patient safety issues related to animal-derived protein extraction, whole ECM proteins are replaced with select functional motifs while preserving the same molecular functionality. In the context of nerve repair, a combination of synthetic peptides present on FN and LN, RGD (Arg-Gly-Asp), and LN-derived YIGSR (Tyr-Ile-Gly-Ser-Arg) and IKVAV (Ile-Lys-Val-Ala-Val) has been shown to be useful (Li et al., 2014). While YIGSR and RGD mediate SC adhesion and proliferation, IKVAV has been shown to support neural differentiation capability and axonal elongation both *in vitro* and *in vivo* (Motta et al., 2019).

Finally, Santiago et al. found that the hADSC adhere preferentially to substrates modified with the IKVAV peptide and less so to YIGSR or RGD (Santiago et al., 2006).

To further investigate the implications of hPL in cell therapy, in this work, we combined hADSC^hPL^ with ECM proteins (LN, FN) in co-culture models with primary DRG neurons in an organotypic tissue explant model. These investigations were conducted with the aim to clarify the effects resulting from a combination of different sera, human ADSC, and two ECM molecules, on primary neurons *in vitro*.

When DRG explants were growing alone (condition 1, without hADSC influence), the two different medium supplements (FBS or hPL) did not influence neurite outgrowth, meaning that the hPL itself, despite its higher GF levels (Guiotto et al., 2020), did not provide significantly higher neurotrophic effects compared with standard FBS (Figure 2) (Palombella et al., 2020). Still, the presence of LN only (DRG alone—condition 1) manifested a meaningful promotion of neurite length and axonal area in comparison with FN or TCPS, independent of the medium supplement used (Figure 2).

When DRG explants were directly grown in contact with hADSC (condition 2), we show a significant difference between the regeneration (longest neurite and axonal extension area) observed for cultures supplemented with hPL and the ones with FBS, in all coating conditions (Figure 3). Remarkably, hPL enhanced the well-known positive effect of hADSC on nerve regeneration (Guo et al., 2017).

When DRG neurons were grown with hADSC-conditioned medium (condition 3), the data showed a significant difference between the hADSC^FBS^ and hADSC^hPL^ secretome where the latter increased neurite length and density, on either ECM-coated surfaces (LN, FN) but not on TCPS. This suggests a significant role of the secretome–matrix–neuron interaction system. Particularly, on LN, primary neurons evidenced the highest response to the hADSC^hPL^ secretome, confirming that LN coating forms part of an ideal environment for regenerating neurons, enabling their response to the neurotrophic effect of the secretome and sustaining axonal elongation (Figure 4) (Armstrong et al., 2007).

**FIGURE 9:**
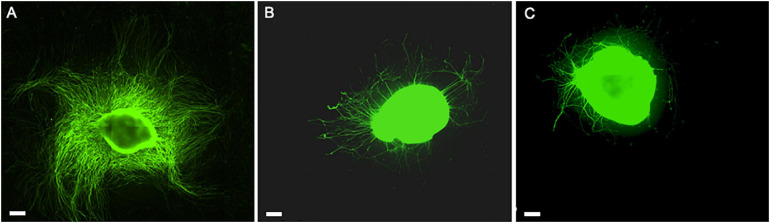
The role of hADSC cell-to-cell interaction and secretome when expanded with the hPL in *in vitro* DRG co-culture model. Direct co-culture of DRG with hADSC^hPL^ on LN-coated surface **(A)**, indirect co-culture of DRG with hADSC^hPL^ secretome on LN-coated surface **(B)**, and DRG alone on LN **(C)**. Scale bar: 500 μm.

The critical role of the interplay between ECM, hADSC, and hPL was evident in the direct co-culture conditions, where we noticed a striking DRG explant outgrowth on LN–hADSC (Figures 5, 9).

Overall, in direct and indirect co-cultures, the neurite outgrowth on LN was significantly increased when compared with the uncoated and even to the FN setting. Interestingly, this effect was only present when hPL was applied as the additive medium during the hADSC expansion steps. Importantly, keeping the hPL supplementation ongoing during the direct co-culture did not seem to further improve neurite outgrowth as we evaluated previously (Palombella et al., 2020).

Indeed, LN directly influences, on the one hand, hADSC adhesion, proliferation, and their neurotrophic properties [supporting GF release (Palombella et al., 2020)]. On the other hand, it creates an environment suitable for regenerating neural cells, prone to be stimulated by the hADSC and their secretome when in co-culture.

hPL stimulates hADSC proliferation and amplifies cell-to-cell interactions and the release of trophic factors synergistically: this effect could be related to the cell proliferation promoted by the hPL itself or to the neurotrophic properties that ADSC acquire when grown with hPL (Palombella et al., 2020).

From a functional point of view, our *in vitro* model can be interpreted as the temporo-spatial sequence of events, which occur after the implantation of a hADSC-loaded neural conduit between the two stumps of a severed nerve. Indeed, while the hADSC secretome (*indirect*) diffuses immediately after the surgery through the local microenvironment, influencing at an early stage (*Time 0*) the nerve growth cone, the hADSC themselves (*direct*) require more time (depending on cell proliferation) to come into contact (*Time 1*) with the axons at the proximal stump, stimulating further elongation.

Not surprisingly, a cumulative effect between the secretome and cell-to-cell interactions emerges from our results. Applying hPL in the pre-expansion steps of hADSC could improve cell performance, reduce the time that lapses (*Time 0*-*Time 1*), support the potential of hADSC to support nerve regeneration therapy at an earlier stage, and lead to an increase in repair when compared with the effect elicited by the same cells grown with FBS.

After peripheral nerve injury, the ECM and, particularly, LN are responsible for providing adequate adaptation signals, through specific integrin-binding receptors, for local cells in order to induce gene expression, metabolic activity, and cell behavior modifications. Similarly, ECM proteins modulate the soluble factor secretion, cellular homeostasis, and cell fate of the hADSC (Midwood et al., 2004; Rozario and DeSimone, 2010; Noro et al., 2013; Pope et al., 2016). Therefore, a LN-functionalized conduit combining ECM scaffold and hADSC^hPL^ could both allow a “neural-oriented” microenvironment for neural cells and a “neural-friendly” behavior of the hADSC themselves. Being expanded with hPL, the stronger proliferation of hADSC^hPL^ amplifies exponentially these benefits.

## Conclusion

To date, this is the first study which investigated the relationship between hADSC, hPL, and ECM: considering our findings, due to the synergic functions, we can hypothesize that a LN-coated scaffold could support hADSC adhesion and proliferation and, in turn, cell activity through GF release and cell–matrix–neuron interactions, maximizing the potential of hADSC therapy.

In light of the remarkable increase of DRG neurite outgrowth obtained *in vitro* on laminin substrates, a combined cell therapy approach coupling hADSC^hPL^ with a LN-functionalized bioartificial conduit will be a matter of further *in vitro* and *in vivo* investigation by our group, to optimize a potential translatable route for peripheral nerve repair.

## Data Availability Statement

The raw data supporting the conclusions of this article will be made available by the authors, without undue reservation.

## Ethics Statement

Ethical review and approval was not required for the animal study because dorsal root ganglia were explanted from euthanized neonatal rats and used as an *in vitro* model for cell culture.

## Author Contributions

MG did the experiments, analyzed the data, and wrote the manuscript. PS conceived the idea and with MR supervised the project and corrected the manuscript. All authors read, critically revised, and approved the final version of the manuscript.

## Conflict of Interest

The authors declare that the research was conducted in the absence of any commercial or financial relationships that could be construed as a potential conflict of interest.

## Figures and Tables

**FIGURE A1:**
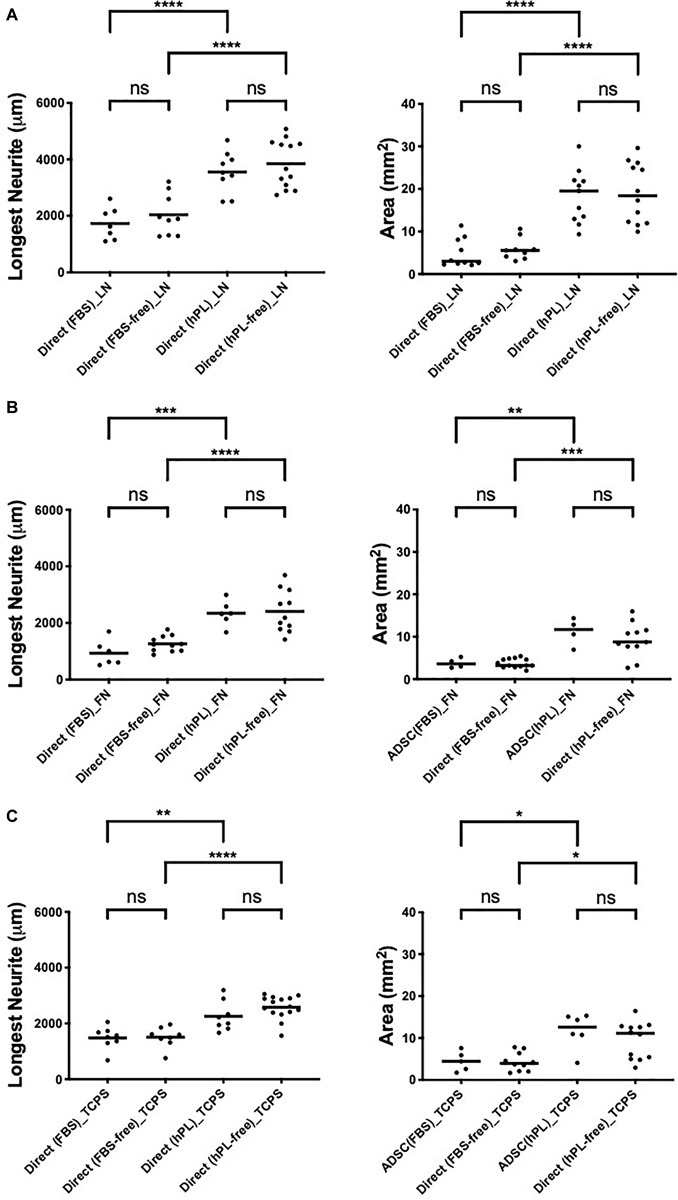
hADSC maintain a neurotrophic effect despite serum deprivation in direct co-culture with primary neurons on LN **(A)**, FN **(B)**, and TCPS **(C)**. Longest neurite and axonal area of direct co-culture on LN **(A)**, FN **(B)**, and TCPS **(C)**. Three biological repeats and at least three technical replicates (DRG) were assessed. One-way ANOVA with Tukey’s multiple comparison tests was used for assessing statistical significance among the examined groups (^∗^*p* < 0.05, ^∗∗^*p* < 0.01, ^∗∗∗^*p* < 0.001, ^****^p < 0.0001). The number of DRG tested corresponds to the number of dots for each condition in the graph.

## Abbreviations

BDNF: brain-derived growth factor
DRG: dorsal root ganglia
ECM: extracellular matrix
FBS: fetal bovine serum
FN: fibronectin
GDNF: glial cell line-derived neurotrophic factor
GMP: good manufacturing practice
GFs: growth factors
hADSC: human adipose-derived stem cells
hPL: human platelet lysate
hADSC^hPL^: human adipose-derived stem cells pre-expanded with hPL-added medium
hADSC^FBS^: human adipose-derived stem cells pre-expanded with FBS-added medium
IKVAV: Ile-Lys-Val-Ala-Val
LN: laminin
MSCs: mesenchymal stem cells
MBP: myelin basic protein
NGF: nerve-derived growth factor
PNI: peripheral nerve injuries
PNS: peripheral nervous system
dADSC: rat ADSC differentiated toward a glia-like phenotype
RGD: Arg-Gly-Asp
SCs: Schwann cells
TCPS: tissue culture plastic
YIGSR: Tyr-Ile-Gly-Ser-Arg.
